# “Going hungry, walking, working, and being cold is hard”: Experiences of Venezuelan migrant parents and caregivers of minors

**DOI:** 10.1371/journal.pone.0329536

**Published:** 2025-08-12

**Authors:** María Pineros-Leano, Beatriz Costas-Rodríguez, Megan M. Taylor, Nancy Jacquelyn Pérez-Flores, Diana Gomez, Natalia Piñeros-Leaño

**Affiliations:** 1 School of Social Work, Boston College, Chestnut Hill, Massachusetts, United States of America; 2 MACONDO Research Team, School of Social Work, Boston College, Chestnut Hill, Massachusetts, United States of America; 3 Department of Clinical Psychology, Carlos Albizu University, San Juan, Puerto Rico; 4 Department of Psychiatry, Washington University School of Medicine, St. Louis, Missouri, United States of America; 5 College of Liberal Arts and Sciences, School of Molecular & Cellular Biology, University of Illinois at Urbana-Champaign, Champaign, Illinois, United States of America; 6 Alberto Lleras Camargo School of Government, Los Andes University, Bogotá, Colombia; University of Alabama at Birmingham, UNITED STATES OF AMERICA

## Abstract

**Background:**

The Venezuelan crisis is one of the largest and most neglected migration crises in the Western hemisphere. Driven by economic, humanitarian, and human rights factors, nearly 8 million Venezuelans have migrated to other countries. Colombia hosts the largest group of Venezuelan migrants worldwide, with approximately 2.9 million Venezuelans residing there. Among these migrants are many Venezuelan parents and caregivers of minors who have resettled in different Colombian cities with their children. This descriptive qualitative study aims to identify their needs and highlight key opportunities for intervention. The perspectives of Venezuelan parents and caregivers of minors were complemented by those of service providers to identify systemic challenges and service gaps, providing a more comprehensive understanding of the population’s needs and priority areas for action. Using the Transnational Theory of Cultural Stress, this study explores the ways in which the experiences of Venezuelan migrant parents before, during, and after migration impact their current needs.

**Methods:**

Using a combination of convenience and probability sampling, we collected semi-structured interviews from 29 Venezuelan parents and caregivers of minors residing in Colombia and 21 service providers who predominantly work with Venezuelan migrants. To analyze the data, we used thematic analysis.

**Results:**

The analysis revealed three major themes: 1) lack of basic necessities forced Venezuelan families to migrate, 2) physical and emotional hardships experienced during the long migration journey, and 3) accumulation of ongoing challenges in Colombia left migrants feeling defeated.

**Conclusion:**

The findings from this study underscore the importance of advancing the United Nations Sustainable Development Goals – including no poverty, reduced inequalities, and good health and well-being – in Venezuelan migrant families.

## Introduction

### Venezuelan context

Venezuela has enjoyed periods of peace and prosperity throughout its history, partly facilitated by its rich oil reserves [1]. In 2016, in response to a fall in oil prices, the Venezuelan government manipulated the national currency to compensate, triggering an economic recession and hyperinflation [2]. Simultaneous political, economic, and humanitarian crises followed, causing Venezuelans from all socioeconomic classes to flee the country [3,4]. To preserve power, the Venezuelan government adopted extreme repressive tactics and perpetrated severe human rights violations against its citizens [5]. These repressive tactics acted as a factor that amplified outmigration [6]. Today, people in Venezuela experience severely limited access to food and medicine [4,7], widespread unemployment [8], and the devastating effects of ongoing hyperinflation, which peaked in 2018 at over 65,000% and currently hovers around 100% [9].

As a result of these crises, 7.7 million people – over 20 percent of Venezuela’s pre-crisis population – have emigrated since 2015 [3,4,10]. Despite being the largest migration in modern Latin American history [11], the Venezuelan exodus ranks among the world’s most neglected and underfunded modern displacement crises [12]. Many Venezuelan migrants have faced substantial personal losses, including losing their homes, jobs, savings, property, and even family members. Under these circumstances, many have decided to endure treacherous journeys to leave Venezuela [13–16].

### Colombian context

Colombia, a middle-income country with a population of over 52 million people [17], shares Venezuela’s longest international land border [8]. Colombia has experienced periods of violence and instability, as Venezuela has, resulting at times in substantial outmigration [1,18–20], as well as high numbers of internally displaced people (IDPs) [21]. In fact, with over 6.8 million IDPs, Colombia has one of the largest internally displaced populations in the world [21], which complicates the country’s ability to effectively manage the inflow of Venezuelan migrants.

### Venezuelan Migrants in Colombia

As of June 2024, approximately 2.9 million Venezuelan migrants were living in Colombia – the largest group of Venezuelan emigrants anywhere in the world [3,10,22]. Within Colombia, the cities of Bogotá and Medellín host the largest number of Venezuelan migrants, followed by Cúcuta, Cali, and Barranquilla [23]. The hardships that Venezuelan migrants encounter are compounded by high levels of discrimination in receiving countries, including Colombia [1,21,23,24]. The United Nations (UN) High Commissioner for Refugees estimates that close to 70% of Venezuelan migrants who live in Latin America and the Caribbean have insufficient access to rights and basic needs in their receiving country, highlighting the dire need for international support, improved policies, and increased access to services for this population [10]. The UN Sustainable Development Goals, including no poverty, reduced inequalities, good health and well-being, provide a compelling justification for addressing these needs [25].

Venezuelan parents and caregivers, or nonparent adults who fill a care-providing, parent-like role for minors in the same household, face unique challenges in Colombia [26–28]. Many of these parents and caregivers (henceforth collectively referred to as ‘parents’) encounter barriers to accessing healthcare, education, and employment, which are often exacerbated by discrimination and lack of legal status [26–28]. Some who find work take on multiple jobs to provide for their children in Colombia as well as family members in Venezuela [27]. Parenting duties also become more difficult in context of tensions between work and caretaking away from community connections they relied on pre-migration [29]. Despite these unique obstacles, the experiences of Venezuelan migrant parents remain underexplored.

Using the Transnational Theory of Cultural Stress, which posits that premigration, transit, and postmigration stressors are interconnected and may impact family functioning and well-being during the resettlement process [29,30], this study aims to identify the needs of Venezuelan migrant parents and caregivers of minors who have resettled in various Colombian cities. Perspectives from Venezuelan migrant parents were complemented by those of service providers to identify systemic challenges and service gaps, providing a more comprehensive understanding of the population’s needs and potential areas for intervention.

## Methods

### Study design and sample

Using a qualitative descriptive research design, participants were recruited using a combination of convenience and probability sampling methods. A sample of 30 Venezuelan parents and 25 providers was predetermined during the design stage. This decision was informed by previous studies that utilized similar sample sizes and successfully achieved data saturation [31,32] and by the availability of resources. Initially, convenience sampling was employed by our research team partnering with Heartland Alliance International (HAI), a U.S.-based humanitarian agency that provides various services in Colombia, including psychosocial support. Recruitment efforts focused on five cities: Bogotá, Tunja, Cali, Barranquilla, and Pasto. The cities were selected to capture a diverse range of participant experiences and were chosen in collaboration with HAI to reflect areas with a large number of Venezuelan migrants where the agency had a physical presence. Once the lists of interested participants were compiled, probability sampling was used to randomly select participants from each city-specific list. This method ensured fairness and reduced selection bias, as participants were randomly chosen from the pool of those who expressed interest.

### Recruitment

To recruit Venezuelan parents, the principal investigator (PI: MPL) provided a flyer and a script in Spanish with detailed information about the study to HAI’s program supervisors, who then relayed the information to program providers. Providers handed out the flyers to Venezuelan parents, and those who expressed interest in participating were included on a list with their contact information (i.e., phone number and email). Supervisors then sent these lists to a designated contact person in the agency, who combined all the information and created city-specific spreadsheets. Information from Bogotá and Tunja was combined because the same office provided services to these two cities. The point person then shared the lists with the PI. The lists of interested Venezuelan parents had 75 eligible individuals, of which 37 were randomly selected using a Microsoft Excel function (S1 Table). The number of participants selected from each city was proportional to the total number of interested participants in that city (i.e., more participants were selected from cities with higher numbers of interested participants). The PI then provided a list to the research assistants (RAs), who contacted potential participants, verified inclusion criteria, and scheduled a time to conduct the interview. Each potential participant was reached on three different occasions and through different methods (e.g., phone call, email). If the potential participant did not reply, the PI randomly selected another participant from the list. The inclusion criteria for Venezuelan parents in the study were as follows: 1) currently living with a child between the ages of 5 and 17, and 2) migrated from Venezuela to Colombia within the last five years (i.e., since 2016). The criteria were established to understand the experiences of recently arrived Venezuelan parents with school-aged children, given the specific needs of this population.

Providers were also recruited in partnership with HAI and from the same cities as Venezuelan parents. First, an introductory email with information about the study was sent out to all providers who predominantly worked with Venezuelan migrants and providers who worked at other humanitarian agencies that provided services to Venezuelan migrants. Providers interested in participating in the study were added to a list of potential participants, which was then shared with the PI. The lists of interested providers had 42 eligible individuals, out of which 25 were randomly selected using a Microsoft Excel function (S2 Table). The number of participants selected from each city was proportional to the number of participants interested in participating from the humanitarian agency and from other community and service agencies. The inclusion criteria for providers were: 1) they work predominantly with Venezuelan migrants and 2) they provide social or health-related services to Venezuelan migrants.

### Procedure

Semi-structured interviews were conducted virtually between October 4, 2021 and February 27, 2022. Three bilingual (English and Spanish) research assistants (RAs) conducted the interviews. Two of the RAs identify as female, and one of them as male. At the time of the study, the RAs had varying levels of education and training. One RA had recently graduated with a Bachelor of Arts in Psychology, another was a first-year student in a Doctor of Psychology program, and the third RA was enrolled in a Master of Social Work program. All RAs had previous experience working with Latin American migrants and received extensive training from the principal investigator (PI: MPL) on the study protocol. The RAs sent reminders to participants one day and one hour before the interview to remind them about the interview. Prior to the first interview, the PI and RAs conducted role-playing activities to practice the interview process. The RAs and PI met weekly to discuss insights, questions or issues that arose during the interviews.

During the first call, the RAs introduce d themselves and the study’s purpose, verified the participants’ inclusion criteria, and scheduled a two-hour appointment for the interview. All procedures with Venezuelan parents and providers were conducted in Spanish. During the interview, the RAs first obtained informed consent from participants and then proceeded to conduct a demographic questionnaire, which included questions on age, gender, and the highest level of education obtained. Among Venezuelan parents, questions on the date of arrival to Colombia and mode of transportation were included. For providers, information on their job title, number of years practicing, and nationality were included.

Following the demographic questionnaire, the RAs collected depression and anxiety symptoms using the validated Patient Health Questionnaire (PHQ-9) and the Generalized Anxiety Disorder (GAD-7) questionnaire, respectively. The RAs then proceeded to conduct the semi-structured interviews. The interview guide for Venezuelan parents asked about their life back in Venezuela, the process of migrating to Colombia, and their life in Colombia (S1 File). The interview guide was pilot tested with a Venezuelan parent, which led to several revisions based on observations and suggestions the participant made. Sample questions include: “Please tell me more about you. What was the hardest thing about the journey for your family? What has been most helpful to your family since arriving in Colombia?.” The providers’ interview guide included questions about their work with Venezuelan migrants and their perspective about the primary issues faced by Venezuelan parents in Colombia. Sample questions include: “What do you think are the biggest stressors for Venezuelan migrant families in Colombia? What are the characteristics of Venezuelan migrant families that help them do well, despite the challenges?”.

### Data analysis

All interviews with Venezuelan parents and providers were recorded. However, one recorder malfunctioned, resulting in a file that could not be played back. Therefore, the interview was excluded from the sample. The recordings were transcribed verbatim through a human transcription company. To verify the transcripts’ accuracy, one RA involved in conducting the interviews listened to all the recordings while reading the transcripts. Three RAs then used thematic analysis to code the data [33]. One of the RAs involved in data collection also participated in the data analysis. The other two RAs were not involved in data collection but had extensive experience coding qualitative data. Each interview was coded by two RAs. One RA coded only data from Venezuelan parents, one coded only data from providers, and one coded data from both, parents and providers.

Following the 6 phases of thematic analysis, in the first phase, the RAs read all the interview transcripts to familiarize themselves with the data [33]. Additionally, the RAs wrote down important ideas that consistently arose from the data. As part of the second phase, the RAs developed a list of initial codes and developed two different codebooks using Dedoose Version 9.0.17 [34]. One codebook was developed for Venezuelan parents and a separate codebook was developed for providers. After developing the codebooks, the RAs systematically coded all the interviews. In phase three, the RAs and the PI met to group the codes into broader themes by clustering the codes that discussed similar experiences. Under this phase and to ensure trustworthiness, the RAs determined the average agreement between coders for each code, which was calculated by identifying the number of times a specific code was assigned out of the total number of interviews [35,36]. Ideally, each code should at least reach an 80% intercoder agreement [35]. When there was less than 80% agreement, the coders discussed code definitions and interpretations until consensus was reached, after which they recoded the interviews [35].

The PI met with the RAs weekly to discuss the codebook and the intercoder agreement. The final intercoder reliability agreement was 88% for the parent data and 82% for the provider data. In phase four, the PI verified that all the coded information under each theme created a coherent pattern. Then, a thematic map of all the themes was developed. Following this, the RAs and the PI checked the thematic map against the transcripts to ensure that the most prominent participant perceptions were encapsulated in the map. Under phase five, the themes were refined, and a description of each theme was created. Also, a title that captured the essence of each theme was identified. As part of the final phase, quotes that exemplified the themes were selected and translated into English using a back-and-forth translation approach [37]. Guided by the Transnational Theory of Cultural Stress, which highlights how stress accumulates throughout the migration journey, the themes were organized into three phases: premigration, transit, and resettlement.

### Research ethics

During the day of the interview, the RAs first read the consent form to the participants and encouraged them to ask questions. Due to the participants’ vulnerable status and their potential apprehension towards signing any paperwork, written consent was not obtained. Instead, the RAs secured oral consent from participants and documented it through audio recordings. This approach was chosen to ensure the participants felt safe and comfortable while still adhering to ethical research standards. All participants were compensated with a grocery store gift card of 40,000 COP (~10 USD) for their time. Participants’ names have been changed to protect participant confidentiality. All activities were reviewed and approved by the Boston College Institutional Review Board under protocol number 21.172.01E and by the Los Andes University Institutional Review Board under protocol number 1274 of 2020. Additional information about inclusivity in global research is available in the S2 File.

## Results

### Sample description

A total of 51 interviews, 30 with Venezuelan parents and 21 service providers, were conducted. Due to a recording malfunction, one parent interview was excluded, resulting in a final sample of 29 parent interviews and 21 provider interviews. The interviews with parents (Table 1) lasted an average of 59 minutes, and those with providers (Table 2) lasted an average of 77 minutes. Most parents were female (97%) with an average age of 37 years. Parents had spent on average, 3.4 years in Colombia. Regarding education level, eight participants (28%) did not graduate from high school, eight (28%) completed high school, and the remaining had attended college or obtained higher levels of education. Most parents were the biological mother of the children they cared for (83%); one was the biological father, three were grandmothers, and one was an aunt. Although most parents migrated with their children to Colombia, a few (n = 6; 21%) had reunited with them after migrating to Colombia. Regarding race, three participants (10%) identified as Afro/Black.

**Table 1:** Parent’s Sociodemographic Information (N = 29).

Characteristic	n	%	*M*	*SD*
Gender	1	3		
Male	28	97		
Female				
Caregiving role	24	83		
Mother	1	3.4		
Father	2	6.8		
Grandmother	2	6.8		
Dual role				
Education	8	28		
Less than high school	8	28		
High school	13	44		
At least some college				
Depressive symptoms	14	48		
None-minimal	8	28		
Mild	6	21		
Moderate	1	3		
Missing				
Anxiety symptoms	11	38		
Minimal	8	28		
Mild	7	24		
Moderate	2	7		
Severe	1	3		
Missing				
Reported having children living in Venezuela	7	24		
Yes	22	76		
No				
Age			36.7	9.7
Years living in Colombia			3.4	1.9
Number of children under their care living in Colombia			2.5	1.3

*Note:* Dual role participants were caring for children in different caregiving roles (e.g., mother and aunt).

**Table 2:** Provider’s Sociodemographic Information (N = 21).

Characteristic	n	%	*M*	*SD*
Gender	6	29		
Male	15	71		
Female				
Occupation	6	28		
Psychologist	5	24		
Community	4	19		
worker	4	19		
Lawyer	1	5		
Social worker	1	5		
Business administrator				
Health				
provider				
Education	2	9.5		
High school	6	28.5		
Some college	8	38		
Bachelor’s degree	5	24		
Master’s degree				
Nationality	19	90		
Colombian	2	10		
Venezuelan				
Age			35.7	9

Most providers were female (71%) with an average age of 35.7 years (Table 2). Most of them were Colombian (90%), but they primarily worked with Venezuelan migrants. A substantial number of them held at least a bachelor’s or a master’s degree (n = 12; 57%). The providers had different occupations including psychologists, community workers, lawyers, social workers, among others.

### Themes

Based on the thematic analysis from the interviews with Venezuelan parents and service providers, three themes were identified. These themes represent the needs and challenges that Venezuelan migrant parents encountered before and during migration, which ultimately impacted their resettlement process in Colombia. These themes include: 1) lack of basic necessities forced Venezuelan families to migrate; 2) physical and emotional hardships experienced during the long migration journey; and 3) accumulation of ongoing challenges in Colombia left migrants feeling defeated (see Table 3). Below we provide an explanation of each theme, along with representative quotes from participants.

**Table 3:** Themes and definitions.

Theme and subtheme	Summary
**Lack of basic necessities forced Venezuelan families to migrate**	Difficulty in accessing food, housing, and jobs due to inflation and lack of access to work opportunities in Venezuela
**Physical and emotional hardships experienced during the long migration journey**	Parents were exposed to harsh conditions in their migrating journey including violence, extreme physical activity, and difficulty navigating transportation systems.
Physical hardships	Described as an acute shortage of food, water, adequate shelter, and rest during the migration journey
Emotional challenges	Nostalgia and grief felt by leaving their lives and loved ones in Venezuela which was exacerbated by parents or children if traveling alone
**Accumulation of ongoing challenges in Colombia left migrants feeling defeated**	Difficulty adapting to life in Colombia as parents are not making enough money, having trouble getting hired, or must send remittances to family in Venezuela.
Sources of income	Disparities in housing expenses compared from the Venezuela way of life and reliance on informal income-generating activities
Housing instability	Scarcity in employment opportunities related to food and housing instability
Degree credentials validation	Diplomas and work experience from Venezuela are not considered valid or difficult to validate in Colombia

#### Lack of basic necessities forced Venezuelan families to migrate.

The first theme captures the conditions that led Venezuelan parents and their families to leave the country. Most participants (n = 27, 93%) expressed experiencing acute hunger in Venezuela, often waiting in line for several hours to receive a few groceries that would only last a few days. Parents described that even when they had some money available, it was not enough to buy food for the whole family due to hyperinflation and the declining value of wages.

In addition to food insecurity, a few Venezuelan parents (n = 9, 31%) also mentioned that their access to medicine and medical attention was severely limited while living in Venezuela. Specifically, they mentioned that it was very difficult to access medical attention due to associated costs and scarcity of providers, and even when they could access it, it was impossible to find and/or afford the prescribed medication. Several parents also mentioned that although they had free housing and public services in Venezuela, these services were ultimately unreliable; for instance, the electricity rarely worked, making it impossible to cook for the family or for the children to do their homework. A parent from Cali shared:

“ The main thing, the shortage, the scarcity of food was terrible because we had to wait in line. Sometimes, we had to wait two or three days to buy bread, flour and that didn’t happen, at least it had a big impact on me and I said that I could no longer continue living in a country where we had to wait in line to eat... Of course, right now, Venezuela is not like this, but it is also difficult because a salary is not enough, now you can get the products but at super high prices and then it is impossible to even return because the work is not sustainable.” Luciana, 29-year-old female living in Cali.

#### Physical and emotional hardships experienced during the long Migration journey.

The second theme encompasses the different hardships parents face d during their migration journey. Several Venezuelan parents described the treacherous journey to Colombia, which spanned between 2 to 4 days and brought numerous challenges and dangers. A total of 23 parents (79.3%) recounted their negative experiences in transit, highlighting the uncertainty of not knowing their exact location at any given time, the lack of support from others, and the constant vulnerability to those who might wish them harm. The physical and emotional toll of the journey was profound, with parents often feeling isolated and exposed. One parent mentioned:

“It was a little traumatic. It’s distressing. First, you go with your loved ones to an unknown place. It’s frustrating to arrive at a transportation terminal where people abuse your needs and exaggeratedly increase the [price of] tickets, where they abuse your need to leave the country to move to another place, where they play with the needs and feelings of people. Being left without money, without knowing what to do, where to go, without knowing anyone - i t’s difficult. It’s really, very frustrating for you. Going hungry, walking, going through work, being cold is hard.” Isabella, 40-year-old female living in Cali.

**Physical Hardships**. One of the most pressing issues parents described was the acute shortage of food and water during the journey. Many parents expressed the intense stress of trying to ensure their children had enough to eat and drink despite the scarcity of their resources. Additionally, parents described the lack of adequate shelter during the journey, with many families spending nights in precarious conditions, unable to find a safe place to rest. One parent from Ipiales summarized the journey as follows:

“I was without cell phone service any of those days, and the hardest thing for me was that I spent almost two days without eating. People usually helped us along the way, people gave us food, but for those two days we hadn’t found anyone who gave us food or who helped us with some money.” Adriana, 38-year-old female living in Ipiales.

All interviewed providers corroborated parents’ accounts, describing their work with Venezuelan migrants who struggled to find sufficient food or rest during the migration journey. F or instance, a provider commented on the hardships that Venezuelan migrants face in transit to Colombia:

“[*Speaking of the journey to Colombia*] Many have been robbed, their belongings and clothes taken...sleeping in the open at night, at risk because there are always unknown groups on the roads, and maybe they will attack them or their children. There are children who are sent by their parents [to travel] with other people.” Alicia, 46-year-old female living in Barranquilla.

**Emotional Hardships**. In addition to the physical challenges encountered during the migration journey, parents also mentioned the emotional toll of leaving behind their lives in Venezuela. They mentioned the nostalgia and sadness they felt around abandoning their country, reflecting on their lives in Venezuela, and the deep and familial connections they left behind in their homeland. Many talked about the grief over leaving loved ones behind in Venezuela, which amplified the negative emotions around migrating to and settling in Colombia. A sense of longing was present as parents reminisced about the positive aspects of their lives in Venezuela. A parent shared:

“The hardest part of the trip for my family was the separation. The separation was very hard from my mom and my brothers. My mom cried a lot because she didn’t [want to] let go of her grandchildren… I don’t know, I guess it’s really very difficult. Right now, well, it’s not that I’ve gotten over it, but I’ve gotten used to it. But at the beginning was very hard, because for example birthdays and Mother’s Day are very hard. And the change of not seeing my mom, of not hugging her, of not being able to tell her that you love her.” Sofia, 40-year-old female living in Barranquilla.

Providers also offered further insight into this aspect of the migration process. Seven providers (33.3%) highlighted the separation of family members during this migration period, with several mentioning that parents or children traveled alone from Venezuela to Colombia, adding another layer of distress to the arduous journey. Providers also mentioned that sometimes husbands would migrate first, leaving the mother and children to travel later. This separation left female parents being the ones facing the brunt of the journey’s challenges alone, intensifying their stress. Nevertheless, providers also emphasized the strength of these female parents who continued to care for and protect their children. For instance, a provider mentioned:

“The majority of people come here from Venezuela with their children. Many pregnant women have come on foot. I met a girl who told me that she was five months pregnant and had two children and came on foot. I said, ‘You are a warrior for having done that!’ But the women come without their husbands...almost always the husband comes here first, and she brings the children after.” Camila, 43-year-old female living in Cali.

#### Accumulation of ongoing challenges in colombia left migrants feeling defeated.

**Sources of Income*.*** The narratives provided by both Venezuelan parents and providers offer insights into the lived experiences of these migrants in Colombia, showing the intricate web of economic challenges and social adversities that began in Venezuela continue on after leaving the country. During the interviews, most (n = 26, 90%) Venezuelan migrant parents describe d a number of economic challenges that stemmed from their transition from Venezuela to Colombia. One of the main economic challenges they faced was the disparity in housing expenses, transitioning from the Venezuelan system where housing costs were subsidized by the government to facing comparatively high unsubsidized rents and utility bills in Colombia that are difficult to afford with the often low and unsteady income that many Venezuelan migrants receive in Colombia:

“The main problems? Let’s say in the work area, sometimes my husband has work and other times he doesn’t find it, so it’s difficult for us because of the rent, the utilities, whether we have to go shopping and it’s a bit complicated for us because it’s only one source of income...” Mariana, 27-year-old female living in Cali.

Several providers also elaborated on the economic challenges communicated by parents, emphasizing the interplay between lack of cultural adaptation, social connectedness, and economic stability. Additionally, they alluded to how the lack of social support networks that Venezuelan migrant families face can exacerbate their sense of vulnerability. For example, providers mentioned that due to reliance on informal income-generating activities, there is a strain on family dynamics where parents often have the dual demands of financial provision and childcare. One provider emphasized the resulting strain:

“It is very difficult to come to a country that is so different from your culture, a country that has many more barriers to caring for children who, if the society wants to harm them, harms them. So, the task of caring for children is very important, and it has to be recognized that it is not easy. And [Venezuelan migrants] are realizing that they have to put their kids on their shoulders, look for a stable job, and they have to sell candy on the Transmilenio [bus system] to earn a few pesos... to work for more than 12 hours to meet your daily expenses is a very difficult task. And many times, they have to take their children to work because they don’t have anyone to leave them with, and obviously they don’t have money to pay someone to watch them. So, it’s a difficult task...” Daniela, 27-year-old female living in Bogota.

Despite Venezuelan parents’ economic challenges faced in Colombia, many sent remittances back to Venezuela and offered economic support to others in Colombia. In our sample, almost 40% of parents (n = 11) mentioned that they provided financial support to family members and friends back in Venezuela. For instance, one parent mentioned:

“Sometimes you worry about the situation there, but if you are sending help or [money], you don’t worry so much about what the stress [in Venezuela] is. It’s a worry that one always has. But as long as one is sending help there, one is calm and has no problems, but sometimes one gets stressed too...” Bianca, 28-year-old female living in Cali.

**Housing Instability.** Most parents (n = 16, 55%) mentioned other barriers that hindered their economic stability in Colombia, including the need to become used to the new economic system, discrimination in job searches, and a scarcity of employment opportunities. Parents mentioned that these difficult circumstances resulted in dire economic situation s, sometimes characterized by food insecurity and housing instability. Parents frequently mentioned that this situation worsened with the COVID-19 pandemic. One parent stated:

“When the pandemic hit, we were left homeless. My daughters and I and my wife. My son was not yet in Colombia when the pandemic [arrived]. This was in May 2020. I was left without a job and I had a cancer diagnosis. Skin cancer, I mean. And I felt, I felt sick, I felt powerless. And then I became unemployed... And well, my wife didn’t work. That night, during the pandemic, we were in quarantine and the owner of the apartment where we were staying [asked us for the apartment] and well, we are not people who stay somewhere, if it’s no longer possible. So, I had to go out on the street. We stayed three days at a friend’s house. I slept in, I was in the living room on a mat and then a friend, she is a community worker, she got us a place in a shelter here in Cali... And we were there for about two months, until the cancer diagnosis disappeared completely. I started to work. And the first month I got paid, I came and rented an apartment right here and we got out of that situation, but it affected us a lot, I would say, a lot.” Diego, 39-year-old male living in Cali.

The interviews with providers resonated with the information provided by parents regarding the housing instability that several Venezuelan migrants face in Colombia. The providers mentioned that the high cost of living, particularly in the main cities, along with low wages, drive food insufficiency and housing insecurity, which can impact the overall well-being of Venezuelan migrant families. A provider emphasize d how the cost of living in Colombia impacted Venezuelan families:

“I think that at a work level, not having a set daily income was stressful. For example, not having enough to pay for a room, or for food for themselves and their children. I arrived from Bogotá one year ago and I saw that many Venezuelans rode the Transmilenio [bus system] selling products or simply panhandling...the pregnant women carrying their children in their arms, and truly in a very desperate situation.” Alejandra, 56-year-old female living in Barranquilla.

**Degree Credentials Validation**. Venezuelan parents also expressed challenges in accessing stable and well-paying job opportunities in Colombia. Parents often mentioned that although several Venezuelan migrants had a professional degree from Venezuela, their diplomas and experience were not valid in Colombia, which forced them to take low-paying or informal jobs. A few parents (n = 4; 14%) described the difficult and lengthy process of getting their degrees from Venezuela validated in Colombia to be able to practice in their professional field. A parent from Cali had this to say about the problems they were facing in Colombia:

“Colombia has given us a lot of strength. Of course, the problems continue to be at the work level, especially at the work level, because many do not have documents, or at least I, am a professional, cannot practice here because I simply do not have my degree written down.” Luciana, 29-year-old female living in Cali.

Providers also emphasized the hurdles that Venezuelan migrants face regarding the lack of recognition of their education credentials in Colombia, which are often not accepted in Colombia’s formal labor sectors. Eight providers (n = 8, 38%) noted that this phenomenon pushes migrants toward informal employment. A provider from Pasto mentioned:

“What’s missing is employment... Colombia is a country where employment is very limited... people struggle to get a job, and especially immigrants. In fact, [if you] come with all the academic training possible... here in Colombia it means nothing. So, it is very difficult, and that’s why we have the employment situation. That is the biggest thing they want - employment. And without employment, without a livelihood, there’s not a good life as a father or mother.” Gabriela, 40-year-old female living in Pasto.

## Discussion

The present study employed the Transnational Theory of Cultural Stress as a guiding framework to understand the experiences of Venezuelan migrants, as reported by parents and service providers across multiple Colombian cities. The Transnational Theory of Cultural Stress provides a unique lens for understanding how stress accumulates over time among Venezuelan parents by considering their experiences before and during migration. These cumulative stressors ultimately shape Venezuelan parents’ needs and challenges during their resettlement process in Colombia. The framework supports our findings through three interconnected themes: 1) lack of basic necessities forced Venezuelan families to migrate; 2) physical and emotional hardships experienced during the long migration journey; and 3) accumulation of ongoing challenges in Colombia left migrants feeling defeated. This study expand s the limited body of literature on the experiences faced by Venezuelan migrants by emphasizing that migration was not a choice but a necessity for Venezuelan parents and families. These results hold potential to inform intervention efforts to improve the quality of life for the largest emigrant group.

The first theme aligns with the premigration phase of the Transnational Theory of Cultural Stress, highlighting how economic collapse and scarcity in Venezuela created chronic stressors that forced Venezuelan parents to migrate. Our findings indicated that Venezuelan parents viewed migration as the only way to survive and offer their children a better future. Participants in the study made the difficult decision to leave everything behind, given the precarious living situation they faced in Venezuela. The experiences described by participants reflect specifically those of parents, who were often motivated to migrate by the desire to provide a safer and more stable environment for their children to grow up in. These findings corroborate previous research conducted among Venezuelan migrants, which has consistently suggested that the deteriorating quality of life among Venezuelans since 2016, has been the driving factor that has pushed them to seek better living conditions in neighboring countries [4,38].

The second theme reflects on the transit-related stressors marked by prolonged uncertainty, exposure to unsafe environments, and a lack of basic necessities such as food, water, and shelter during the migration journey. These conditions not only posed immediate threats to physical health but also triggered emotional trauma, particularly for those traveling with children or separated from family members. The emotional burden of navigating unfamiliar terrain, facing exploitation, and enduring harsh weather conditions compounded the stress that was present during the premigration phase. These findings underscore how the transit phase is not merely a logistical transition but a critical period of vulnerability that can have lasting effects on the coping mechanisms that Venezuelan parents and families can rely on during resettlement. These findings corroborate previous research that has identified similar in-transit experiences of food insecurity, with some estimates suggesting that a staggering 74% of migrants experience moderate to severe levels of food insecurity during the migration journey [39,40].

In addition to food insecurity, Venezuelan parents mourned having to leave behind their nuclear or extended family, along with their familiar customs and culture. Our participants mentioned that during the journey to Colombia, they faced emotional challenges and often reminisced about their pre-crisis lives in Venezuela. Although the emotional turmoil may wane as Venezuelan migrants begin to settle into new places [41,42], nostalgia for their home country often stays with them well after resettlement [4,43,44]. These findings indicate the importance of supporting Venezuelan parents during the transit phase by providing mental health screenings [45] and mental health support [46,47], which can prove effective during their resettlement process. In fact, previous studies have found elevated depressive symptoms of up to 80% among recently arrived Venezuelan migrants in Colombia [4,43] and the United Stated [4], suggesting that mental health screenings should be a public health priority in order to ensure a smooth integration process.

The third theme in our study aligns with the postmigration stressors described in the Transnational Theory of Cultural Stress, which emphasizes how challenges encountered after arrival to the new country can compound earlier stress and hinder successful integration. Venezuelan parents in our study reported experiencing significant economic hardship, including housing instability and underemployment, which undermined their expectations of a better life in Colombia. These stressors were not only financial but also emotional, as they often led to feelings of defeat and disillusionment.

In the present study, several of the participants were unable to secure employment in their field once they arrived in Colombia, despite having at least a technical degree from Venezuela. Instead, they turned to informal work, a trend that has been similarly found in broader research on Venezuelan migration [48]. In fact, previous research among Venezuelan migrant adults in Colombia has indicated that approximately 92% are informally employed [49]. Informal employment, while often the only available option, is associated with unsteady income, lack of benefits, occupational hazards, and increased mental and physical distress [48–51]. These conditions can exacerbate the psychological burden migrants already carry from their premigration and transit experiences. The lack of degree validation further compounds these challenges. Migrants often face a lengthy and bureaucratic process to have their qualifications recognized, which delays or prevents their entry into formal employment [48,50,51]. Streamlining credential recognition could significantly reduce economic vulnerability and improve housing stability, while also promoting dignified and sustainable integration into the host society.

### Implications

The findings from this study suggested that cumulative stressors experienced during premigration, and transit phases ultimately influence the wellbeing of Venezuelan families during their resettlement process in Colombia. Despite Colombia sharing a common language and some cultural traditions with Venezuela, many participants in our study emphasized the cultural disconnect they experienced after resettlement. These differences—ranging from social norms to institutional expectations—highlight the need for culturally responsive interventions. Mental health providers could consider incorporating strategies to address the ongoing economic stressors, potentially including financial counseling, job placement assistance, and support groups for migrants facing economic hardship. Additionally, it is essential for providers to address trauma that may stem from the experiences that Venezuelan families faced in their home country. Mental health initiatives should prioritize the integration of migrants into the host community by fostering social support networks and enhancing access to essential services, such as healthcare and education. While some migrant families may not have experienced trauma, it is crucial for providers to be vigilant of the potential impacts of such experiences.

The results of this study also shed light on the importance of addressing issues such as the regularization of Venezuelan migrants, housing instability, economic fragility, and credential validation, all of which act as deterrents to financial stability. The combination of persistent nostalgia for Venezuela and the socio-economic challenges Venezuelan migrants face in Colombia warrants multifaceted, empathetic responses from researchers and policymakers to improve their quality of life.

### Limitations

Despite this study’s contributions, it is also essential to acknowledge its limitations. First, the findings are constrained by the timing of data collection, as migration experiences are dynamic and can evolve over time. The data were collected in 2021 and 2022, which may not fully capture the ongoing and changing nature of Venezuelan migrants’ current experiences in Colombia. Second, the selection of cities for participant recruitment presents a limitation. Although cities such as Cúcuta and Medellín host large populations of Venezuelan migrants, they were not included in the study. Third, the diverse backgrounds and experiences of Venezuelan migrants suggest that needs can vary widely, making it challenging to capture the full range of circumstances. Future studies could expand recruitment to additional cities to better reflect the heterogeneity of this population. Lastly, the inclusion criteria for service providers did not require a minimum duration of experience working with Venezuelan parents, which may have impacted the depth and breadth of the information gathered. Additionally, the assessment to which providers primarily worked with Venezuelan migrants was based on self-report. Future research could benefit from incorporating more objective and nuanced measures to verify the extent of providers’ work with specific populations. Despite these limitations, the study offers valuable insights into the needs of Venezuelan migrants in Colombia and contributes meaningfully to the existing literature.

## Conclusion

The Venezuelan migration crisis remains one of the most urgent yet under-researched humanitarian emergencies in the region. The present study contributes to the growing body of literature by centering the voices of Venezuelan migrant parents and Colombian service providers across multiple cities, with the goal of identifying needs, systemic challenges, and service gaps to inform potential areas for intervention.

The findings reveal a continuum of hardship: from the scarcity of basic necessities that compelled families to leave Venezuela, to the emotional and physical toll of the migration journey, and the persistent economic struggles faced during resettlement in Colombia. These insights underscore the pressing need for comprehensive, culturally responsive support systems that address the full migration experience—from departure to integration.

By aligning with the United Nations Sustainable Development Goals, this study advocates for actionable strategies to reduce poverty, hunger, inequality, and health disparities among Venezuelan migrants. In doing so, it lays a foundation for future research, policy development, and cross-sector collaboration aimed at mitigating the long-term impacts of this crisis and improving the well-being of the Venezuelan diaspora.

## Supporting information

S1 Table Selection of Venezuelan parents by city of residence. (PDF)

S2 Table Selection of providers by city of residence. (PDF)

S1 File Interview guide. (PDF)

S2 File Inclusivity in global research. (PDF)
