# Supplementary material for: “Going hungry, walking, working, and being cold is hard”: Experiences of Venezuelan migrant parents and caregivers of minors
Source: PLoS One. 2025 Aug 12;20(8):e0329536. doi: 10.1371/journal.pone.0329536 (PMC12342323; doi:10.1371/journal.pone.0329536)
Supplement: S1 Table — (PDF) [file pone.0329536.s001.pdf]

S1 Table

Table 1. Selection of Venezuelan parents by city of residence

City	Total number of interested individuals	Total number of randomly selected participants
Barranquilla	8	4
Pasto	11	5
Bogotá	3	2
Tunja	7	3
Cali	46	23
Total	75	37
