# Supplementary material for: “Going hungry, walking, working, and being cold is hard”: Experiences of Venezuelan migrant parents and caregivers of minors
Source: PLoS One. 2025 Aug 12;20(8):e0329536. doi: 10.1371/journal.pone.0329536 (PMC12342323; doi:10.1371/journal.pone.0329536)
Supplement: S2 Table — (PDF) [file pone.0329536.s002.pdf]

S2 Table

Table 2. Selection of providers by city of residence

City	Total number of interested individuals	Total number of randomly selected individuals
Barranquilla	10	6
Pasto	9	5
Bogotá	7	5
Tunja	2	1
Cali	14	8
Total	42	25
