# Supplementary material for: “Going hungry, walking, working, and being cold is hard”: Experiences of Venezuelan migrant parents and caregivers of minors
Source: PLoS One. 2025 Aug 12;20(8):e0329536. doi: 10.1371/journal.pone.0329536 (PMC12342323; doi:10.1371/journal.pone.0329536)
Supplement: S1 File — (PDF) [file pone.0329536.s003.pdf]

S1 File

Interview guide

SECTION A. First, I would like to ask about you.

1. Please tell me more about you. When did you arrive to Colombia? How long have you been in [current city]?
2. Tell me about your family.
    a. *Sub-questions:* Who is part of your family? Who from your family is with you in Colombia? Are there people in your family who are still in Venezuela? Are there people in your family who have migrated to other countries?

SECTION B. Now I'd like to talk a bit about what life was like before you [and your family] left Venezuela.

1. What was your life like in Venezuela?
    a. *Sub-question:* What was it like for your family?
2. What were the biggest problems you faced in Venezuela?
    a. *Sub-question:* What were the biggest problems for your family?
3. When you had problems, where did you go or who did you talk to?
4. Why did you decide to come to Colombia?
5. Who made the decision to leave Venezuela?
6. What was the hardest thing for you about leaving Venezuela?

SECTION C. Now I'd like to hear more about your journey to Colombia. [If participant traveled by plane, only ask questions 1, 2, and 7]

1. How did you travel to Colombia (e.g. walking, bus)?
2. Who did you travel with?
3. For how long were you traveling before you reached [current city]? Did you stay in any other cities in Colombia along the way?
4. What was the hardest thing about the journey for you?
5. [If participant traveled with children] What was the hardest thing about the journey for your children or other children you were with?
6. Was anyone or anything especially helpful to you along your journey? Who or what?
7. How did you feel when you arrived to [current city]?

SECTION D. Now I'd like to talk more about what your life is like now. About the things that are difficult and the things that help.

1. Can you please describe your living situation? Please don't use anyone's name, specifically.
    a. *Sub-questions:* Do you live with family members? Friends? People you have met since arriving to Colombia?
2. What are the biggest problems you face here in Colombia?
    a. *Sub-question:* How are these problems similar or different to other Venezuelans in Colombia?
3. What do you do to make those problems better? Is there a place you go to or someone you talk to?

4. Think about the times when you feel good or happy. What is happening in those moments?
5. Who do you spend most of your time in Colombia? Do you have a group of Venezuelans here in Colombia who you spend time with?

SECTION E. Now I'd like to talk more about what life for your children and other Venezuelan children is like now.

1. What are the biggest problems that Venezuelan children face here in Colombia?
2. What do children do to make those problems better? Is there a place they can go or someone they usually talk to?
3. What helps children do well or feel happy, despite the current challenges?
4. What would make things better for Venezuelan children who have migrated to Colombia?

SECTION F. Now, I'd like to talk with you about how Venezuelan families are doing here in Colombia.

1. What has been most helpful to your family since arriving to Colombia?
2. What are the biggest challenges your family has faced in Colombia that you did not have in Venezuela?
3. How has your relationship with your children changed since you migrated to Colombia?
4. How has your relationship with other family members changed since you arrived to Colombia?
5. If you were designing a program that would help Venezuelan families here in Colombia, what would it be like?
    a. *Sub-questions*: Would it be better for this program to be delivered with many parents/families at once or should it target one family at a time? Should it be at home or at a center? What would be an ideal number of sessions?

SECTION G. Now, I'd like to talk with you about how Venezuelan caregivers are doing here in Colombia.

1. How does it feel being a parent/caregiver in Colombia? How does this differ from when you were in Venezuela?
2. What helps you do well as a parent or caregiver, despite the current challenges?
3. What kind of help would make parenting or caregiving easier?

SECTION H. I'd like to ask you a few questions about mental health and Venezuelan migrants.

1. How has mental health been affected since you migrated to Colombia?
    a. *Sub-questions:* Do people behave differently than they used to? Do they communicate differently with others? Does mental health affect their work? Does mental health affect their ability to have fun or play?
2. Do mental health concerns affect the functioning of Venezuelan migrants in Colombia?
3. What do Venezuelan migrants do when they are worried about their mental health or the mental health of friends or family members?
    a. *Sub-questions:* Where do they go? Who do they talk to?

SECTION J. The COVID-19 pandemic has changed many people's lives since it began. I'd like to ask you a few questions about if and how it has affected your and your family's life.

1. How do you feel the pandemic has affected you [and your family] in Colombia?
    a. *Sub-questions:* Has it affected your ability to make money? Your living situation? Your ability to access basic life necessities like food, medical care, child care, education, or other?
2. Have your plans for the future changed since the pandemic started? If so, how?
3. How has the pandemic affected your mental health?
4. How has the pandemic affected the mental health of other Venezuelan migrants?
5. From where or whom do you get most of your information about COVID-19?
    a. *Sub-questions:* Are [were] the rules and regulations related to the lockdown clear to you? How closely were you able to abide by the regulations?
6. Do you think the situation of Venezuelan migrants in Colombia has been adequately considered during the COVID-19 crisis? If so, tell me what you think is going right. If not, tell me what you think is going wrong or missing from the response.

CLOSING. I have just one more question for you.

1. Is there anything important about Venezuelan families and their experience here in Colombia that I haven't asked you and you would like to share?

Thank you for taking the time to speak to me today. We appreciate your time and knowledge.
